# CGENet: A Deep Graph Model for COVID-19 Detection Based on Chest CT

**DOI:** 10.3390/biology11010033

**Published:** 2021-12-27

**Authors:** Si-Yuan Lu, Zheng Zhang, Yu-Dong Zhang, Shui-Hua Wang

**Affiliations:** 1 School of Computing and Mathematical Sciences, University of Leicester, Leicester LE1 7RH, UK; sl672@le.ac.uk; 2 Shenzhen Key Laboratory of Visual Object Detection and Recognition, Harbin Institute of Technology, Shenzhen 518055, China; darrenzz219@gmail.com or; 3 Department of Computer and Information Science, University of Macau, Macau 999078, China

**Keywords:** computer-aided diagnosis, transfer learning, convolutional neural network, feedforward neural network, extreme learning machine, graph neural network

## Abstract

**Simple Summary:**

This study proposes a new COVID-19 detection system called CGENet, based on computer vision and chest computed tomography images. First, an optimal backbone selection algorithm was proposed to determine the best backbone network for the CGENet adaptively. Then, we introduced a novel graph embedding mechanism to fuse the spatial relationship into the feature vectors. Finally, we chose the extreme learning machine as the classifier of the proposed CGENet to boost the classification performance. The proposed CGENet was evaluated on a public dataset using 5-fold cross-validation and compared with other algorithms. The results revealed that the proposed model achieved state-of-the-art classification performance. In all, the CGENet can be an effective and efficient tool that can assist COVID-19 diagnosis.

**Abstract:**

Accurate and timely diagnosis of COVID-19 is indispensable to control its spread. This study proposes a novel explainable COVID-19 diagnosis system called CGENet based on graph embedding and an extreme learning machine for chest CT images. We put forward an optimal backbone selection algorithm to select the best backbone for the CGENet based on transfer learning. Then, we introduced graph theory into the ResNet-18 based on the *k*-nearest neighbors. Finally, an extreme learning machine was trained as the classifier of the CGENet. The proposed CGENet was evaluated on a large publicly-available COVID-19 dataset and produced an average accuracy of 97.78% based on 5-fold cross-validation. In addition, we utilized the Grad-CAM maps to present a visual explanation of the CGENet based on COVID-19 samples. In all, the proposed CGENet can be an effective and efficient tool to assist COVID-19 diagnosis.

## 1. Introduction

COVID-19, named by the World Health Organization, is a serious and contagious lung pneumonia caused by the coronavirus. The epidemic began in the winter of 2019 and spread worldwide shortly after. Most countries had to carry out lockdowns to control the epidemic, resulting in great economic loss. Isolating the confirmed COVID-19 cases is an effective way to control the pneumonia because the coronavirus can be transmitted among human beings by droplets. Therefore, accurate and early diagnosis is the primary step.

Currently, the real-time reverse transcription-polymerase chain reaction (rRT-PCR) test is the prevailing method for this pneumonia [1]. On the other side, medical imaging techniques can achieve good visualization on the development of COVID-19 in the human body, such as computed tomography (CT). There is massive information in chest CT images that can be beneficial for clinical diagnosis. Manual analysis of the chest CT images, which also suffer from low reproducibility, is a heavy burden for radiologists. Over the last decade, computer-aided diagnosis (CAD) has become a heated research topic. CAD systems can implement image analysis automatically and accurately to generate predictions of cases, which is one of the directions of smart healthcare. With advanced deep learning models and algorithms, CAD systems are now obtaining better performance. Recently, researchers and practitioners presented several CAD methods for COVID-19 diagnosis via medical images.

Sun, et al. [2] proposed their CAD COVID-19 detection model called AFS-DF for chest CT images. Initially, local features were extracted based on CT images. Afterwards, they trained a forest to implement high-level representation learning as well as adaptive feature selection. Their method was evaluated on a dataset with multiple lung diseases, including COVID-19. The accuracy of the proposed algorithm was 91.79%. Roy, et al. [3] suggested utilizing deep learning models to analyze automatically lung ultrasonography (LUS) images. They modified a spatial transformer network to predict the scores of the LUS frames and localize the potential lesions simultaneously. They also put forward an aggregation mechanism to generate video-level predictions. Their model achieved satisfactory results in the experiments. Oh, et al. [4] proposed an automatic COVID-19 classification system based on chest X-ray images. A fully convolutional DenseNet was employed to generate contour patches from X-ray images. They then extracted small patches from these contour images and used these patches to train a group of pre-trained ResNets. Finally, the predictions of the original samples were generated based on the majority voting method. Ouyang, et al. [5] discovered that COVID-19 CT datasets were usually class-imbalanced, so they proposed a dual-sampling algorithm to handle this problem. For classification, they presented an attention-based network. The 5-fold cross-validation (CV) was used to evaluate their method, and the accuracy was 87.5%. Wang, et al. [6] presented a CAD method to implement classification and lesion localization simultaneously based on 3D CT images. Firstly, a UNet was employed for 3D image segmentation. Then, they constructed a 3D deep model named DeCoVNet, and trained it to predict the infectious probabilities. Finally, the potential lesions were localized based on the activation maps and a weakly supervised method. Wang, et al. [7] developed their COVID-19 classification method using a COVID-Net. As the CT samples in different datasets were visually different, they tried to improve the generalization ability of COVID-Net using CT datasets from different sources. A feature normalization mechanism was implemented on the top of the COVID-Net, and the objective function was improved with a contrastive learning strategy. The performance of their model is promising. Yu, et al. [8] tested a bunch of pre-trained deep CNN models with traditional machine learning classification algorithms. Four famous backbone models were employed to extract image features, and five classic classification algorithms were trained to identify COVID-19. Zhou, et al. [9] developed a chest CT simulator to generate more samples from real cases to handle the data scarcity problem in training deep models for the diagnosis of COVID-19. A deep convolutional architecture was proposed for segmentation and classification which converted the tedious 3D CT segmentation into 2D segmentation. Angelov and Soares [10] provided a large COVID-19 CT dataset and presented an explainable model for classification. Their method yielded an accuracy of 97.38%. Alam, et al. [11] provided a CAD system using feature fusion for COVID-19 classification. They first extracted two different features sets using a gradient-based method and a convolutional neural network (CNN). Then, a VGG network was trained for classification. They also employed a watershed algorithm to segment the samples. Gupta, et al. [12] integrated a bunch of on-the-shelf CNN models to construct their InstaCovNet-19 network which was trained to classify COVID-19 in X-ray images. Their system achieved state-of-the-art classification performance in the experiments. Jain, et al. [13] trained an Xception network with their chest CT images to detect COVID-19. Experimental results suggested that their system produced an accuracy of 97.97%. Kc, et al. [14] fine-tuned a pre-trained DenseNet-121 to detect COVID-19. It is reported that only 62% of the total parameters in the DenseNet-121 were re-trained in their simulation. Kumar, et al. [15] put forward a federated learning method to detect COVID-19 using datasets from different sources with privacy protection. They invented a normalization approach to deal with the variance of the samples. Then, they trained a capsule network for every local dataset and generated their global model using federated learning. Rahimzadeh, et al. [16] exploited ResNet-50V2 as the backbone model and proposed a feature pyramid approach to improve its classification performance for COVID-19 classification. Sadre, et al. [17] presented a protocol to validate COVID-19 CAD systems and summarized some weaknesses in current methods. Shankar and Perumal [18] employed a Gaussian filtering method for pre-processing of chest CT images. Afterward, they extracted deep learning features using an InceptionV3 and computed handcrafted features with the linear binary pattern algorithm. An entropy-based fusion method was employed to fuse the two types of features. Finally, a multi-layer perception was trained for classification. Zebin and Rezvy [19] used a generative adversarial network for data augmentation in order to obtain more COVID-19 samples for training. An EfficientNet pre-trained on the ImageNet dataset was employed as the backbone. For fine-tuning, they had only to update the parameters in the fully connected layers. Zhou, et al. [20] employed three deep CNNs for representation learning from CT images, and used softmax as the classification method. The final predictions were obtained using the relative majority voting. Wang, et al. [21] used a deep graph neural network to classify COVID-19 samples from normal controls in chest CT images. Their model was evaluated on a private dataset and produced promising classification results. Ozturk, et al. [22] proposed a DarkCovidNet with 17 convolutional layers to detect COVID-19 in X-ray images. For binary classification (COVID-19 versus No-findings), their model yielded a sensitivity of 95.13 based on 5-fold cross-validation.

The above methods have produced promising results, but we hold the view that the classification performance can be further boosted. Many of these methods either employed deep models or traditional machine learning algorithms for classification. However, deep models usually require big datasets to train, and the training also demands a long time (usually several days) to converge. On the other side, traditional machine learning algorithms are dependent on the distribution of handcrafted features, which can be a challenge because the images contain massive information. Furthermore, the relationship among the samples can be used for classification, which is often neglected. To this end, we proposed a novel CGENet (COVID-19 Graph Extreme learning machine Network) based on the graph neural network and extreme learning machine (ELM) to detect COVID-19 in chest CT images. We put forward an optimal backbone selection algorithm to choose the best backbone model of the CGENet and leverage transfer learning to fine-tune it on the CT images. The relationship between the feature vectors in the latent space can be harnessed for the classification because the image features are closely related to their labels. It is believed that samples of the same labels should share similar features. We employed the k-NN algorithm for graph embedding in the CGENet based on the feature vectors extracted from the CT scans to leverage this relationship among the feature vectors. Finally, an ELM serves as the classifier in the CGENet to identify the samples as COVID-19 or non-COVID-19. The proposed CGENet is evaluated on a public dataset based on a 5-fold CV. The results of the experiments reveal that the CGENet yielded state-of-the-art classification performance. Meanwhile, the human readability of the deep networks is a significant topic in the development of CNN models, so the Grad-CAM is employed to offer a visual explanation of the CGENet. The regions of interest from the CGENet can be seen in the Grad-CAM maps based on the COVID-19 chest CT images. The contributions of this paper are summarized as follows:

1. An optimal backbone selection algorithm was presented to determine the backbone of the proposed CGENet.
2. A novel graph embedding mechanism was proposed to leverage the relationship among the image feature vectors.
3. An ELM served as the classification algorithm for the CGENet.

The arrangement of the rest of this manuscript is shown below. The chest CT dataset in the experiments is presented in Section 2. Section 3 concerns the detailed presentation of the proposed CGENet. Section 4 provides the experimental results and discussion. The conclusion and future research directions are given in Section 5.

## 2. Materials

A public chest CT dataset named SARS-CoV-2, was employed to evaluate the classification performance of the CGENet, which is available on the Kaggle website (available: https://www.kaggle.com/plameneduardo/sarscov2-ctscan-dataset (accessed on 7 August 2021)).

**Figure 1:**
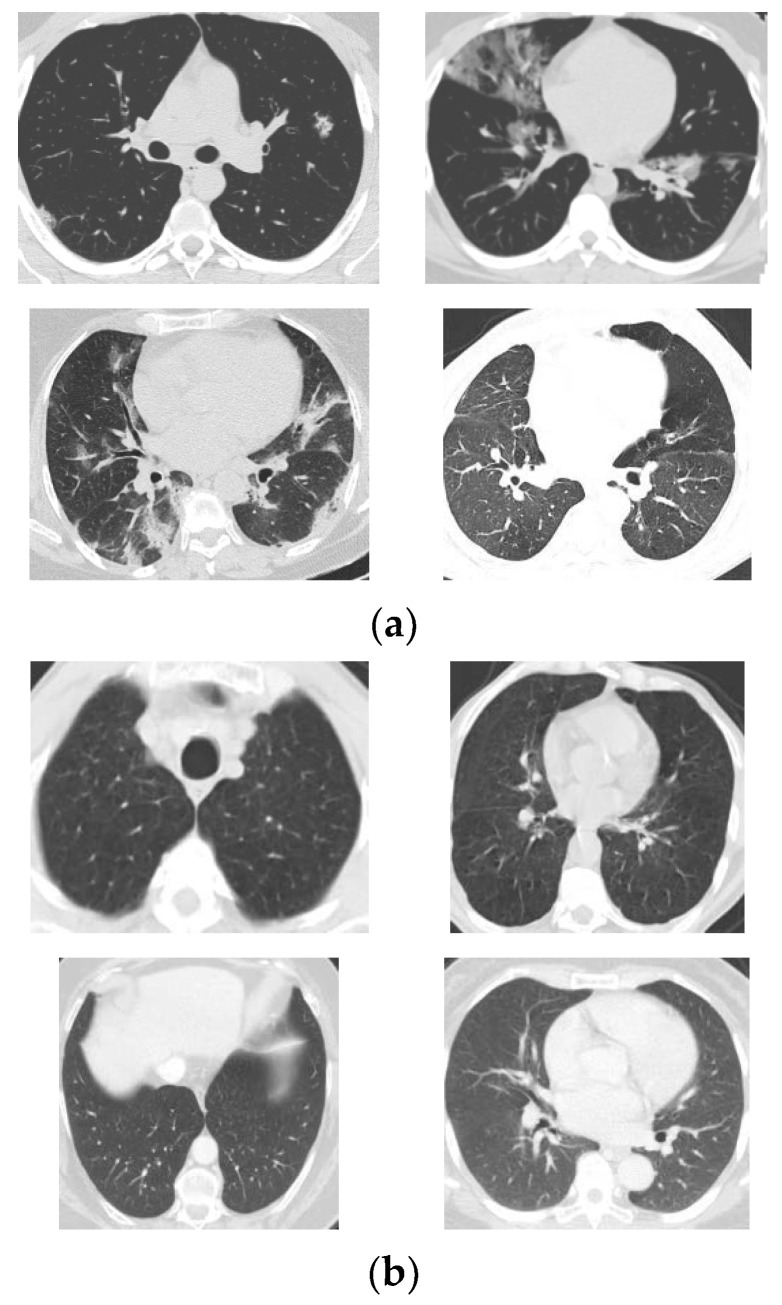
Random CT scans in the SARS-CoV-2 dataset ((**a**) COVID-19 samples, (**b**) Non-COVID-19 samples).

All the CT scans are from patients in Brazil. There are 1252 COVID-19 positive samples and 1230 non-COVID-19 ones in the public dataset. The non-COVID-19 samples mean that the patients are not affected with COVID-19 but other pulmonary diseases. The resolution of the CT scans varies from around 100 × 100 to around 500 × 500. Therefore, all the images need to be resized to the dimension of the input layer of the CGENet before training and testing. More details of the SARS-CoV-2 dataset are presented in the works of literature refs. [10,23]. Some random CT scans in the dataset are listed in Figure 1.

## 3. Methodology

**Figure 2:**
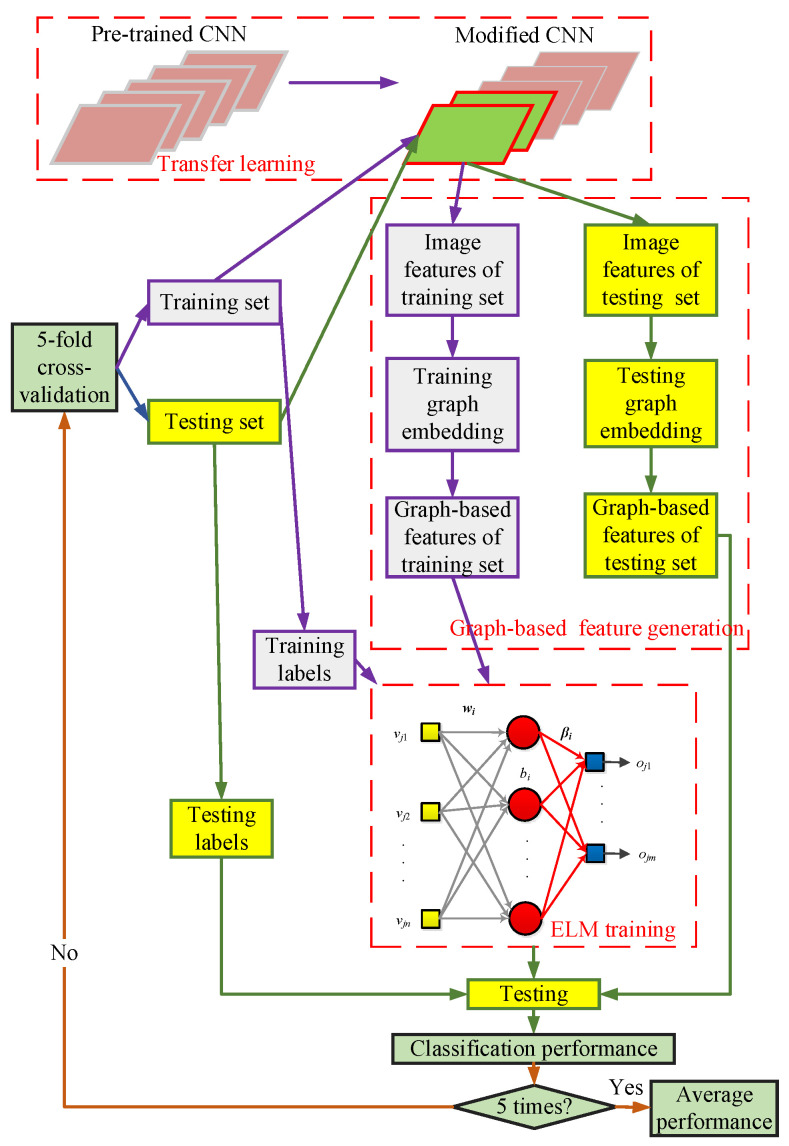
Pipeline of the proposed CGENet.

This study used chest CT images and put forward a new and straightforward COVID-19 detection model called CGENet. The pipeline of the proposed CGENet is demonstrated in Figure 2. Initially, a pre-trained model was employed as the backbone of the CGENet. Using transfer learning, the ResNet-18 was modified and fine-tuned on the COVID-19 dataset. Then, the image features could be obtained from the intermediate layer of the CNN model. To determine the optimal backbone, an optimal backbone selection algorithm was proposed. Afterward, a feature graph was generated based on the feature set using the *k*-NN algorithm to obtain the graph-based image features. Finally, an ELM was utilized as the classifier of the proposed CGENet to identify the graph-based image features. The 5-fold cross-validation was utilized to obtain the out-of-sample classification performance of the CGENet. More detailed analysis and discussion of the CGENet are provided below.

### 3.1. Backbone Network Selection

Deep learning has caused profound effects in both academia and industry over the last decade. CNN models are the most prevailing method to deal with image processing tasks, and the best performance on the famous ImageNet dataset is refreshed every year. Since the success of the AlexNet [24], an ocean of deep models has been invented, such as DenseNet [25], ResNet [26], VGG [27], MobileNet [28], EfficientNet [29], etc. In the meantime, CNN models have been applied in face recognition, machine translation, and even autonomous driving.

The outstanding performance of these deep CNN models revealed the powerful image classification and segmentation ability. However, dedicated graph cards and big labeled datasets are usually required to train CNN models from scratch. Meanwhile, the training demands too much time, which sometimes lasts for several days. Big labeled datasets posed another challenge to using deep models because manual labeling is a tedious job, and some datasets require expertise to label, such as medical image datasets. All these challenges made it difficult to train CNNs from scratch, so researchers were prone to utilize a transfer learning approach to apply pre-trained CNNs for specific tasks.

It is inevitable to cause overfitting if a deep CNN is trained from scratch on a small dataset because there are massive parameters in the CNN. Transfer learning handles it in a different way. CNN backbones are often pre-trained on the ImageNet dataset, which is a large labeled image dataset including 1000 categories. Hence, these pre-trained models can already classify 1000 categories of images accurately, which means that they can generate useful image features. These models’ outstanding feature extraction capability can be transferred to another dataset that can be very different from the ImageNet dataset. The ImageNet dataset is usually treated as the source domain (SD), while the other dataset is called the target domain (TD). Although there are differences between the two domains, such as the categories of samples and the distribution of samples, there can be similarities between the two domains in the latent feature space. In transfer learning, a deep CNN model’s complex high-level representation generation ability is transferred from the SD to the (TD) by fine-tuning the network on the target dataset. In practice, the target datasets are usually much smaller than the ImageNet dataset, and the results are usually satisfactory. With transfer learning, the pre-trained deep model can be transferred from the big dataset to a relatively small dataset, and users only need to fine-tune the model for several epochs. Therefore, overfitting can be avoided. The fine-tuning is also much faster on the small target dataset, which can be run on an ordinary laptop or personal computer.

We proposed an algorithm to select the optimal pre-trained backbone network for the proposed CGENet. First, famous deep CNN models were obtained, including AlexNet, ResNet-18, ResNet-50, MobileNetV2, and EfficientNet, all pre-trained on the ImageNet dataset. The structures of the five CNN models have to be modified to the COVID-19 dataset. There are only COVID-19 and non-COVID-19 samples in the public dataset, so the dimension of the output vector is set to be 2. Additionally, an extra fully connected layer with 256 nodes was added to serve as the feature layer. The added feature layer is also beneficial to mitigate the gap in dimensions of the adjacent layers. The optimizer is Adam. After fine-tuning the modified CNNs with the COVID-19 training set, the optimal backbone model can be obtained by comparing the average testing accuracy based on a 5-fold CV. The pseudo-code of the optimal backbone model selection algorithm is presented in Table 1. In the simulation, the CGENet from ResNet-18 yielded the best accuracy. Therefore, ResNet-18 was the best backbone. As a result, the image features can be extracted conveniently from the training set and testing set using the optimal backbone model.

### 3.2. Graph Embedding

The image features obtained by the backbone model were from the individual CT scans. However, the relationship between the feature vectors in the latent space can be harnessed for the classification because the image features are closely related to their labels. It is believed that samples of the same labels should share similar features. Therefore, we proposed the employment of a graph to embed the relationship of the feature vectors in the latent feature dimension using the *k*-NN algorithm. Given a set of image feature vectors as

(1) $$V_{F}={\left[v_{1},v_{2},v_{3},\ldots ,v_{N}\right]}^{T}\in {\mathbb{R}}^{N\times D}$$

Wherein *N* and *D* denote the number of images in the dataset and the dimension of the feature vector, respectively, the graph of the feature vectors can be generated using the *k*-NN algorithm based on the Euclidian distances between the vectors. Then, the matrix describing the distances of the feature vectors **DM** and the matrix describing the adjacent relationship of the feature vectors **AM** can be obtained by

(2) $$DM\left(i,j\right)=\|v_{i}-v_{j}\|,\;1\leq i,j\leq N$$

(3) $$AM\left(i,j\right)=1,\;\mathrm{if}\;v_{j}\in neighbors\left(v_{i}\right),\;1\leq i,j\leq N\;$$

In which **DM** and **AM** are initialized as zero matrixes. Afterward, the normalized adjacent matrix can be expressed as

(4) $$\hat{AM}=Deg^{-\frac{1}{2}}\left(AM+I\right)Deg^{-\frac{1}{2}}$$

Wherein **I** represents the identity matrix and **Deg** the degree matrix:

(5) $$Deg\left(i,j\right)=\{\begin{array}{c} k,\mathrm{if}\;i=j\;\\ 0,\;\mathrm{if}\;i\neq j\; \end{array},\;1\leq i,j\leq N$$

Eventually, graph embedded features can be computed by

(6) $$V_{\mathrm{GE}}=\hat{AM}\cdot V_{F}$$

**Figure 3:**
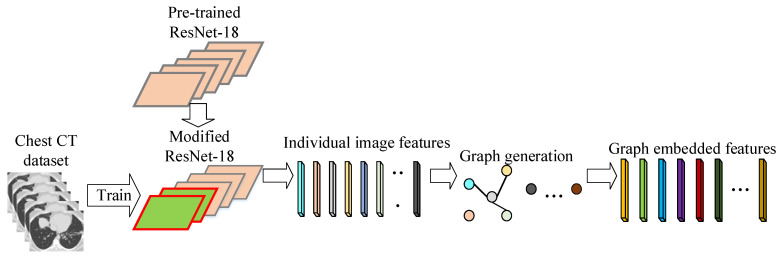
Flowchart of the graph embedding.

A flowchart of the graph embedding is shown in Figure 3. The graph embedded features can be generated for the training set and testing set similarly, which is consequently used to train and test the ELM in the CGENet.

### 3.3. Selection of Classifier of CGENet

The training of deep models is tedious, and the obtained solution of parameters may not be optimal so that the classification performance can be improved. Therefore, we proposed to use randomized neural networks to boost the classification performance of the CGENet further because they can be trained extremely fast but produce promising classification results. Two types of randomized neural networks, extreme learning machine (ELM) and random vector functional-link (RVFL), were selected as the classifier of the CGENet and were compared to obtain the best one.

**Figure 4:**
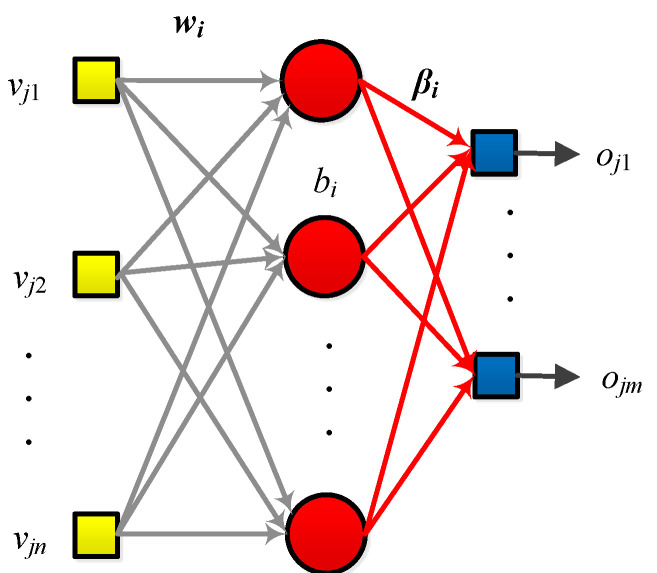
Structure of an ELM.

The ELM belongs to the randomized neural network [30]. The ELM architecture is provided in Figure 4. The training steps of ELM are easy to implement. Given the training set as

(7) $$TS=\left(V_{\mathrm{GE}},Y\right)$$

in which **Y** represents the labels of the training samples, and an ELM with $\hat{N}$ hidden nodes, first calculate the activation matrix **AcM** of the hidden layer by

(8) $$AcM=\overset{\hat{N}}{\underset{i=1}{\sum }}g\left(w_{i}v_{j}+b_{i}\right),j=1,\ldots ,N$$

in which *g*(*x*) stands for the sigmoid function. The weights $w_{i}$ and biases $b_{i}$ of the hidden layer are initially assigned with random values. Then, the objective for training is to show that the predictions from the ELM are equal to the actual labels of the training samples:

(9) AcM·β=Y

Therefore, the output weights β are calculated using pseudo-inverse:

(10) $$\beta =AcM^{†}Y$$

in which $AcM^{†}$ is the pseudo-inverse of **AcM**. With only simple steps, the ELM is trained. Meanwhile, only the dimension of the hidden layer $\hat{N}$ is the pre-defined parameter, so ELM requires less manual intervention.

**Figure 5:**
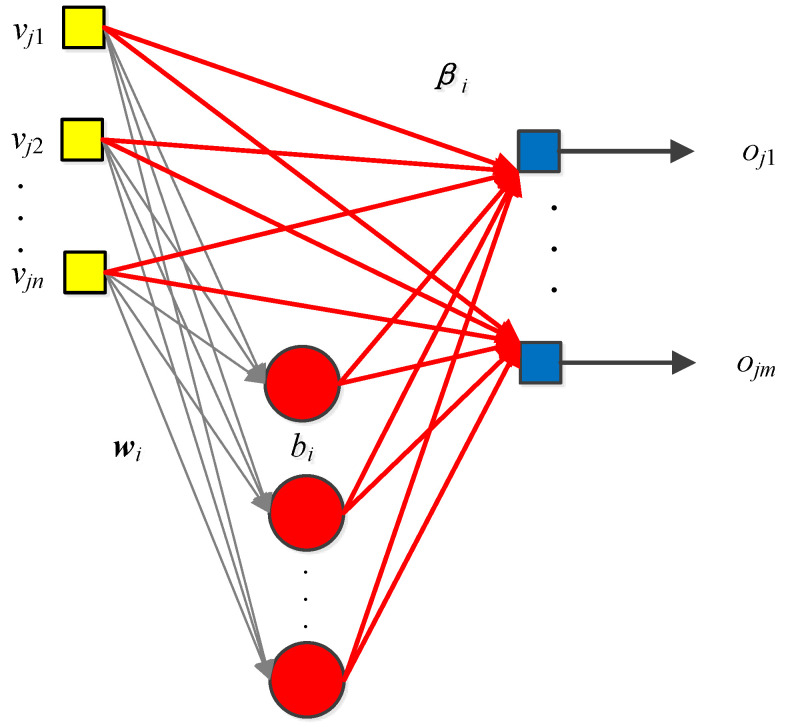
Structure of an RVFL.

The architecture of the RVFL is different from the ELM because there is a shortcut directly from the input space to the output layer, shown in Figure 5 [31,32]. The training of the RVFL is similar to ELM. The only difference lies in the calculation of the input matrix of the output layer $\hat{AcM}$, which can be obtained by concatenation:

(11) $$\hat{AcM}=concat\left(AcM,V_{\mathrm{GE}}\right),$$

where the **AcM** represents the activation matrix of the hidden layer, and **V**_GE_ the graph embedded feature set. The weights and biases in the hidden layer are randomly initialized, and the output weights can be obtained by pseudo-inverse using the $\hat{AcM}$ and **Y**.

To obtain a better classifier of the CGENet among ELM and RVFL, we proposed an optimal classifier selection algorithm. We evaluated the CGENet with the two different classifiers based on 5-fold cross-validation and selected the better one based on the overall accuracy. The pseudo-code of the optimal classifier selection method is shown in Table 2. The experiment results showed that the CGENet using the ELM as its classifier performed better, so we chose to employ the ELM as the classifier in the CGENet.

## 4. Experimental Results and Discussion

The proposed CGENet was implemented based on MATLAB R2021a with the pre-trained models. The evaluation experiments were carried out based on 5-fold cross-validation. The source code of our model is available at https://github.com/SiyuanLuLSY/CGENet (accessed on 15 December 2021).

### 4.1. Hyper-Parameters

The values of hyper-parameters in the proposed CGENet are presented in Table 3. In the first stage, the mini-batch size was set to be 24, considering the GPU memory and the size of our COVID-19 dataset. The max epochs were set to be 2 to avoid overfitting, and the learning rate for fine-tuning was 1 × 10^−4^, which belongs to a conventional value. The value of *k* in the *k-*NN algorithm was 4, which means that every feature vector was linked with its four nearest neighbors to generate a graph. Finally, the dimension of the hidden layer in the ELM was 512, which is twice the dimension of the graph embedded features.

### 4.2. Classification Results of the CGENet

The results of our CGENet on the public dataset using 5-fold CV are illustrated in Table 4. The entire time of training and testing for 5-fold CV was 676.32 s, which was much less than that of training deep models from scratch. It can be observed that our CGENet achieved over 97.50% for all the five widely used evaluation measurements, and the worst value was also more than 96.00% for all the 5 folds. The results suggested that our CGENet is an effective and robust CAD system, which can assist radiologists in their diagnosis.

### 4.3. Performance of the CGENet with Different Backbone Models

The backbone model played a vital part in the performance of the CGENet. However, there is no strict rule for the backbone model selection because it is highly data-dependent. Therefore, we experimented using different backbone models, and the average performance based on 5-fold cross-validation is presented in Table 5. It can be seen that the CGENet with AlexNet performed the worst while the other four backbones achieved close results. ResNet-18 yielded marginally better classification performance than ResNet-50, MobileNetV2, and EfficientNet. Therefore, ResNet-18 was selected as the best backbone for the CGENet.

### 4.4. Performance of the CGENet with Different Graph Embedding Strategies

The number of neighbors in the *k-*NN algorithm is an important hyper-parameter for the graph generation. We experimented with different values of *k*, and the simulation results from the 5-fold CV are presented in Table 6. The CGENet with different graph embeddings produced very close classification results, and the graph embedding with four neighbors performed marginally better. The CGENet with larger values of *k* would require more training time, so the optimal value of *k* was chosen as 4.

### 4.5. Performance of the CGENet with Different Classifiers

We tested the proposed CGENet with ELM and RVFL as the classification algorithms. The performance results based on the 5-fold CV are given in Table 7. We discovered that the CGENet with ELM performed slightly better than that with the RVFL in terms of accuracy. Meanwhile, the computational cost for the ELM was lower than the RVFL because the concatenated matrix in the RVFL training is bigger in size. As a result, we selected the ELM for classification in the CGENet.

### 4.6. Explainability of the CGENet

**Figure 6:**
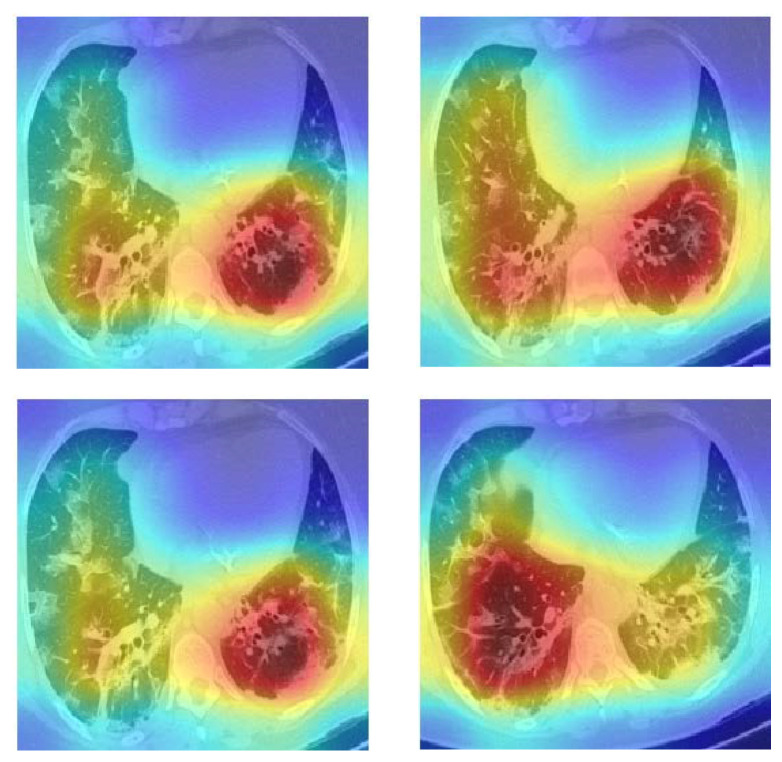
Grad-CAM heat maps of COVID-19 cases.

We wanted to find out why the CGENet could produce accurate predictions, so the Grad-CAM was employed to visualize the model’s attention [33]. We provide several heat maps of the COVID-19 examples in Figure 6. The CGENet paid more attention to the red and yellow regions and disregarded the blue regions when making predictions. We can see that the CGENet can roughly locate the potential lesion areas to make accurate predictions.

### 4.7. Comparison with Other Existing Methods

The proposed CGENet was also compared with the other five existing methods. The comparison is listed in Table 8. The public dataset in evaluating the CGENet was also employed in the experiments of xDNN and SepNorm + Contrastive. It could be found that the proposed CGENet was better than xDNN as well as SepNorm + Contrastive for F1-score, sensitivity, and accuracy. Although the accuracy and precision of DarkCovidNet were slightly better than the CGENet, the CGENet produced the highest sensitivity, specificity, and F1-score. Additionally, the DarkCovidNet was trained for 100 epochs on a dataset smaller than our dataset, but our max epoch was only 2 to avoid overfitting. The comparison revealed that the CGENet was accurate for the diagnosis of COVID-19, which was contributed to by transfer learning and graph embedding.

## 5. Conclusions

This study presented a new explainable COVID-19 CAD method called CGENet. An optimal backbone selection method was put forward to determine the best backbone for the CGENet based on transfer learning. The CGENet further harnessed a novel graph embedding mechanism to fuse the spatial relationship among the image feature vectors. For the classification, an ELM was trained. The CGENet was evaluated on a public chest CT dataset. Experiment results suggested that the proposed model achieved good classification performance in COVID-19 recognition, comparable to five existing methods.

For future research, we will continue to gather more samples in terms of both quantity and category because the situation in real-world clinics is more complex. A patient may be affected by multiple diseases and therefore, multi-class classification should be investigated. In addition, the ELM can be further optimized, and we will try other classification models. Another challenge is that the COVID-19 related signs on chest CT images show poor specificity as they can be seen in many other pulmonary diseases. We aim to test the proposed model in a real-world scenario and improve the generalization ability in the future because the proposed CGENet was only evaluated on a single dataset, and it may not produce the same good classification results on other datasets.

## Figures and Tables

**Table 1:** Pseudocode of the optimal backbone model selection algorithm.

Step1:	load the COVID-19 dataset.
Step2:	load the pre-trained backbone models, including AlexNet, ResNet-18, ResNet-50, MobileNetV2, and EfficientNet.
Step3:	modify the structure of these backbones based on the labels of the COVID-19 dataset using ELM.
Step4:	train these models and test them based on 5-fold cross-validation.
Step5:	compute the average testing accuracies of the 5 backbones.
Step6:	output the optimal backbone model which yielded the highest average testing accuracy.

**Table 2:** Pseudocode of the optimal classifier selection algorithm.

Step 1:	load the COVID-19 dataset.
Step 2:	load the ResNet-18.
Step 3:	modify the structure of the ResNet-18 based on the labels of the COVID-19 dataset using ELM and RVFL, respectively.
Step 4:	train the two models and test them based on 5-fold cross-validation.
Step 5:	compute the average testing accuracies of the two models.
Step 6:	output the CGENet with the better classifier, which yielded the highest average testing accuracy.

**Table 3:** Hyper-parameter settings in the CGENet.

Method	Hyper-Parameter	Value
Transfer learning	Batch size	24
	Max epochs	2
	Learning rate	1 × 10^−4^
*k-*NN for graph embedding	*k*	4
ELM and RVFL	$\hat{N}$	512

**Table 4:** Results of the CGENet based on 5-fold CV (unit: %).

	Accuracy	Sensitivity	Specificity	Precision	F1-Score
Fold 1	98.18	97.63	98.76	98.80	98.21
Fold 2	97.18	96.47	97.93	98.01	97.23
Fold 3	96.57	96.41	96.73	96.80	96.61
Fold 4	98.59	100.00	97.23	97.20	98.58
Fold 5	98.39	99.19	97.60	97.61	98.39
Average	97.78	97.94	97.65	97.68	97.80

**Table 5:** Results of the CGENet using different backbones based on 5-fold CV.

Backbones	Accuracy	Sensitivity	Specificity	Precision	F1-Score
AlexNet	75.45	78.87	73.15	69.96	73.81
ResNet-18	97.78	97.94	97.65	97.68	97.80
ResNet-50	97.50	97.83	97.18	97.20	97.51
MobileNetV2	97.02	97.37	96.73	96.72	97.03
EfficientNet	97.50	97.70	97.33	97.36	97.52

**Table 6:** Results of the CGENet with different graph embeddings (unit: %).

Value of *k*	Accuracy	Sensitivity	Specificity	Precision	F1-Score
3	96.90	96.25	97.62	97.68	96.95
4	97.78	97.94	97.65	97.68	97.80
5	96.94	96.76	97.15	97.20	96.97
6	96.90	96.98	96.85	96.88	96.92

**Table 7:** Results of the CGENet using different classifiers based on 5-fold CV.

Classifiers	Accuracy	Sensitivity	Specificity	Precision	F1-Score
ELM	97.78	97.94	97.65	97.68	97.80
RVFL	97.30	97.99	96.64	96.65	97.31

**Table 8:** Comparison with other existing approaches (unit: %).

Methods	Accuracy	Sensitivity	Specificity	Precision	F1-Score	Dataset
xDNN [10]	97.38	95.53	-	99.16	97.31	SARS-CoV-2
SepNorm + Contrastive [7]	90.83	85.89	-	95.75	90.87	SARS-CoV-2
FGCNet [21]	97.14	97.71	96.56	96.61	97.15	Private dataset
DarkCovidNet [22]	98.08	95.13	95.3	98.03	96.51	Mixed public datasets
Dual-Track Learning [1]	-	86.00	-	89.60	87.80	Private dataset
CGENet (ours)	97.78	97.94	97.65	97.68	97.80	SARS-CoV-2

## Data Availability

Not applicable.
